# Psychopathological aspects in patients with diabetes

**DOI:** 10.1192/j.eurpsy.2023.730

**Published:** 2023-07-19

**Authors:** S. Trifu, A. I. Vasile, A. Ciubară

**Affiliations:** 1 Neurosciences; 2 Doctoral School, University of Medicine and Pharmacy Carol Davila, Bucharest; 3 Psychiatry, Dunarea de Jos University of Medicine and Pharmacy, Galati, Romania

## Abstract

**Introduction:**

This paper highlights the mutual influences and the relationships between the variables of type II diabetes, the type of psychiatric conditions, the administered treatment schemes, the imaging examinations and the impact on the functioning and quality of life of the patients.

**Objectives:**

Analysis of the influences between diabetes and psychiatric disorders, studying the relationships between the variables of type 2 diabetes, the type of psychiatric disorders, treatmentregimens, imaging examinations and the impact on the functioning and quality of life of patients.

**Methods:**

Psychiatric interview, Hamilton, Reisberg and Rosenberg scales, laboratory analysys

**Results:**

Patients with pre-existing diabetes, psychiatric disorders led to deterioration of its evolution, documented by HbA1c values, treatment schedule, frequency of diabetic emergencies; the increased frequency of psychotic phenomena (hallucinations and delusional ideas) is directly proportional to the number of diabetic emergencies; patients with uncontrolled, long-term diabetes have higher scores on HAM-D and Reisberg scales, while patients with controlled diabetes have higher scores on the self-esteem and quality of life scales; in patients with taste or odor disorders, cortical atrophy may be seen on CT examination, elevated HbA1c levels and the presence of polyneuropathy; alcohol consumption, smoking, high cholesterol levels, determine the advancement of diabetic complications, and these in turn correlate with higher scores on the HAM-D scales, Reisberg; patients who show large variations in blood glucose in the first days after hospitalization are those who exhibit irritability, irritability, nervousness and heteroaggression at the time of hospitalization;

**Conclusions:**

65% patients with depressive disorders, 10% - with organic personality disorder, 25% - with affective disorders and 30% associated with cognitive impairment. The duration of diabetes mellitus, glycosylated hemoglobin levels and the presence of diabetic complications is directly related to the HAM-D and Reisberg scores; and inversely proportional to quality of life scores and self-esteem.

**Disclosure of Interest:**

None Declared

